# Extirpation of the Uterus for Complete Inversion

**Published:** 1845-02

**Authors:** 


*Extirpation of the Uterus for Complete Inversion.*—M. Velpeau has recently extirpated the uterus in a case of inverson, the result of labour, which had existed four years, and threatened the life of the woman through the continued hæmorrhage which it occasioned. After bringing down the uterus by means of double hooks, (erignes,) ligatures were passed through the base of the tumor which it formed, and it was then excised. From the account given of the operation, it is rather difficult to understand how the ligatures were applied. In another case in which M. Velpeau operated, three months ago, it appears that the base of the tumor was traversed with two ligatures, and they were then tied circularly. Owing, however, to the gradual retraction and ascension of the divided surfaces, the ligatures slipped off. In this case, therefore, it is stated, that the ligatures were placed from one side to the other and united in front, so as to approximate the divided surfaces, as in the operation for hare-lip. The patient died of peritonitis a few days after the operation.

M. Velpeau has operated by excision on a woman who recovered. He has lost another patient by hæmorrhage, and has met with three other cases of complete inversion; he has therefore seen six cases in all.

*London Lancet*, from *Gazette des Hospitaux.*

